# Remaining Tooth Structure and Prognosis of Restored Endodontically Treated Teeth: A Systematic Review

**DOI:** 10.7759/cureus.94406

**Published:** 2025-10-12

**Authors:** Saja K Al-Meshal, Aqeel M AlismaiI, Tamim A Alfalah, Abdulaziz S AlRashidi, Hadeel J Al-Mubarak, Waleed K Almohammadi, Abdulelah A Al-Jaban, Abrar M Almutairi, Talal K Alotaibi, Abdulrahman S Alamari

**Affiliations:** 1 Dentistry, Majmaah University, Al-Majmaah, SAU; 2 General Dentistry, King Faisal University, Al-Ahsa, SAU; 3 General Dentistry, Ministry of Health, Dammam, SAU; 4 College of Dentistry, University of Hail, Hail, SAU; 5 College of Dentistry, Riyadh Elm University, Riyadh, SAU; 6 Dentistry, Ministry of Health Saudi Arabia, Dammam, SAU; 7 Dentistry, King Abdulaziz Medical City Riyadh, Riyadh, SAU; 8 Dentistry, Princess Nourah Bint Abdulrahman University, Riyadh, SAU; 9 College of Dentistry, Taif University, Taif, SAU; 10 Family Dental Medicine, Riyadh Second Health Cluster, Ministry of Health, Riyadh, SAU

**Keywords:** cuspal coverage, endodontically treated teeth, ferrule effect, remaining tooth structure, tooth survival

## Abstract

The prognosis of endodontically treated teeth is closely tied to the amount and configuration of remaining coronal tooth structure. This systematic review compared the survival of permanent endodontically treated teeth without fracture (fracture-free survival) restored with non-cuspal-coverage direct resin composites versus full/partial-coverage crowns, with a focus on ferrule presence/continuity, axial wall integrity, and the extent of surface loss as modifying factors. Following PRISMA guidance, the review included randomized trials, prospective and retrospective cohorts, and clinical studies from five databases with manual citation tracking; studies involving mature permanent teeth and reporting survival were eligible. Five studies fulfilled the inclusion criteria; the number of teeth considered by the studies ranged from 105 to 420 teeth. Short-term outcomes were uniformly high, with several studies reporting 100% survival at ≤36 months for both crowns and direct composites. In cohorts that used longer follow-up periods of approximately 8-10 years, survival rates were generally higher for crowned teeth, especially when structural loss was extensive. Where reported, stratification by remaining tooth structure (RTS) suggested that teeth with one to three surfaces lost and preserved axial walls performed well with adhesively bonded direct composites, whereas teeth with four to five surfaces lost or disrupted ferrule benefited from cuspal coverage. Risk-of-bias assessment was generally low to moderate, though some studies showed concerns related to deviations from intended interventions or outcome measurement. Overall, crowns and direct composites both achieved high survival rates in the short term; however, the overall evidence indicates a long-term advantage for cuspal coverage in structurally compromised teeth. Evidence certainty is limited by heterogeneity, small numbers, and incomplete reporting of remaining tooth-structure variables, underscoring the need for standardized RTS reporting and longer, well-controlled studies.

## Introduction and background

The restoration and long-term prognosis of endodontically treated teeth are multifactorial; however, a consistent finding across different studies is that the preservation of coronal tooth structure is the most influential determinant of survival and fracture resistance. Endodontic treatment itself does not inherently render teeth brittle; rather, biomechanical integrity is compromised by a combination of securing access and pre-existing structural loss [1,2]. Laboratory and clinical investigations show that residual coronal dentin volume is directly proportional to fracture resistance. For example, restored maxillary anterior teeth with intact axial walls can tolerate substantially higher failure loads than teeth that have only half of their axial walls [3]. In maxillary premolars, a strong positive correlation (r = 0.72) has been demonstrated between remaining coronal dentin surface area and fracture strength: teeth with all axial walls intact withstand loads near ~1,380 N, whereas loss of palatal and proximal walls reduces resistance to ~345-398 N [4]. Comparable patterns have been reported for maxillary incisors, where removing one or both proximal walls significantly diminishes fracture resistance, and fiber post-placement helps restore strength when coronal structure is limited [5]. These findings underscore that both the amount and the spatial distribution of dentin, particularly the axial walls and the presence of a ferrule, are critical to stability under function.

The ferrule effect, defined as a circumferential band of sound dentin typically 1.5-2 mm in height above the preparation margin, is a cornerstone of post-endodontic restoration design. This structural collar resists lever forces, attenuates stresses associated with post placement, and redirects functional loading, thereby protecting the root from vertical fracture [6]. Accordingly, teeth with ≥2 mm of axial wall height demonstrate superior resistance compared with those without a ferrule [7]. Importantly, ferrule requirements include not only height but also dentin thickness and circumferential continuity; dentin thickness of <1 mm, especially interproximally, elevates failure risk, and the location of missing walls influences outcomes [8,9]. Clinical and epidemiological observations support these experimental data. A Swiss registry review reported that molars with <30% residual coronal volume were approximately three times more likely to be extracted at follow-up than molars with ≥30% remaining and that endodontically treated teeth lacking cuspal coverage exhibited roughly six-fold higher failure rates than those restored with full-coverage crowns over 9-10 years [10]. Furthermore, retrospective observations on molars suggest increased fracture and microbial leakage when teeth are left unrestored after root canal therapy.

The role of posts should be viewed primarily as retention for the core rather than reinforcement of the tooth. When sufficient coronal dentin remains to support the core and crown, routine post placement may confer little benefit and can introduce fracture risk; accordingly, posts are best reserved for situations with inadequate coronal structure [11]. Fiber posts, with an elastic modulus closer to dentin, are associated with fewer catastrophic failures, particularly under incisal loading [12]. In vitro evidence also indicates that in the absence of one or more axial walls, fiber-post placement can boost survival cycles and reduce irreparable fracture modes [13].

From a clinical standpoint, treatment planning should prioritize conservation of residual tooth structure. The main principles include maintaining remaining axial walls and marginal ridges to optimize a circumferential ferrule of 1.5-2 mm with adequate dentin thickness, using minimally invasive preparation strategies that avoid unnecessary enlargement, and considering crown lengthening or orthodontic extrusion to establish a ferrule in the case of insufficient structure. Fiber posts should be employed judiciously to retain core material rather than as a substitute for an absent ferrule [11]. Overall, the prognosis of restored endodontically treated teeth hinges foremost on preserving coronal tooth structure. Ferrule presence, axial wall integrity, and residual dentin volume exert a greater impact on fracture resistance and long-term survival than the specific post system or core material, reinforcing the value of minimally invasive access, conservative post-endodontic preparation, and strategic restorative planning to maximize structural resilience and clinical success. Accordingly, this review is structured around the following PICO question: In permanent teeth requiring post-endodontic restoration (Population), does the amount and configuration of residual coronal tooth structure (Intervention), compared with reduced or absent axial walls/ferrule (Comparison), influence tooth and restoration survival, fracture incidence, and extraction rates (Outcome)?

This systematic review aims to determine the influence of the amount and configuration of remaining coronal tooth structure on the prognosis of endodontically treated permanent teeth restored with either direct resin composites or indirect full/partial-coverage restorations. The review compares fracture-free tooth survival, restoration survival, catastrophic/root fracture, and extraction rates between crowns/onlays and direct composites. In addition, it examines how these outcomes vary by ferrule height and continuity, axial wall count, number of surfaces lost, tooth type, and post type across short-term (~24-36 months) and longer-term (~8-10 years) follow-up.

## Review

Methods

Study Design and Objective

This systematic review aims to assess and compare the prognosis of endodontically treated permanent teeth restored with either non-cuspal-coverage direct resin composites or full-/partial-coverage crowns, with particular emphasis on the influence of the amount and configuration of remaining coronal tooth structure on fracture-free survival and related outcomes. No protocol was prospectively registered; a predefined internal plan specified the research question, eligibility criteria, outcomes, and analysis approach, and any deviations are reported in this manuscript.

PICOS Eligibility Criteria

The following PICOS Eligibility Criteria were used:

Population (P): human permanent endodontically treated teeth of any type (incisors, canines, premolars, molars).

Intervention (I): non-cuspal-coverage direct resin composite restorations.

Comparison (C): full- or partial-coverage indirect restorations (e.g., crowns, onlays).

Outcomes (O): primary: fracture-free tooth survival at ~24-36 months and ~8-10 years. Secondary: restoration survival, catastrophic/root fracture, extraction, and complications (e.g., loss of retention, re-treatment).

Study design (S): randomized controlled trials, prospective cohorts, retrospective cohorts (clinical human studies). In vitro/ex vivo studies, immature/primary teeth, case reports/series without a comparator, and studies without clinical follow-up were excluded.

Eligibility Criteria

The population comprised permanent endodontically treated teeth of any tooth type (incisors, canines, premolars, or molars) restored with direct resin composites without cuspal coverage or with indirect full- or partial-coverage restorations (e.g., crowns or onlays). Comparative study designs were eligible, including randomized controlled trials, prospective cohorts, and retrospective cohorts; single-arm studies were considered only if they contributed contextual survival data and could not drive comparative conclusions. Only studies reporting clinical follow-up outcomes relevant to survival or fractures were included. Exclusion criteria were in vitro or laboratory studies, studies on immature or primary teeth, case reports/series without a comparator, and reports that did not provide clinical follow-up. Studies restricted solely to anterior teeth were not prioritized unless they directly informed the comparative question; such studies were considered qualitatively where applicable.

Definition of Remaining Tooth Structure

The remaining coronal tooth structure was operationalized a priori using commonly reported clinical descriptors. When available, data were extracted for axial wall count (0-1, 2, 3, or 4 walls), number of surfaces lost (one to three versus four to five), ferrule height (0, <1 mm, 1-2 mm, >2 mm) and circumferential continuity (yes/no), dentin thickness (particularly interproximal), tooth type, and the presence and type of post (none, fiber, or metal). When studies used alternative but mappable definitions (e.g., percentage of residual coronal volume), these were recorded verbatim and aligned to the closest remaining tooth structure (RTS) categories for subgroup analysis where feasible.

Outcome

The primary outcome was fracture-free tooth survival at the clinically relevant time horizons of approximately 24-36 months and 8-10 years. Secondary outcomes included restoration survival, catastrophic/root fracture, extraction risk, and complications such as loss of retention or need for re-treatment. Where both tooth-level and restoration-level data were reported, tooth-level outcomes were prioritized.

Information Sources and Search Strategy

A comprehensive electronic search was conducted in PubMed, Web of Science, ScienceDirect, Scopus, and MEDLINE, covering all available publications from database inception through September 2025. The search was limited to clinical human studies published in English. Search strings were tailored to each database using controlled vocabulary and keywords related to endodontically treated teeth, fracture resistance/survival, resin composite restorations, full-coverage crowns, remaining/ lost tooth structure, and prognosis. An example PubMed string was as follows: (“endodontically treated teeth” OR “root canal treated teeth”) AND (“fracture resistance” OR “survival rates”) AND (“resin composite restoration” OR “full-coverage crowns”) AND (“tooth structure loss” OR “remaining tooth structure”). Grey literature and trial registries (e.g., ClinicalTrials.gov, WHO ICTRP) were screened, and reference lists of included studies were hand-searched to identify additional records. All records were deduplicated prior to screening.

Study Selection

Titles and abstracts were screened for relevance, followed by full-text assessment against the eligibility criteria. Two reviewers independently and manually screened all titles/abstracts and full texts, and discrepancies were resolved through discussion.

**Figure 1:**
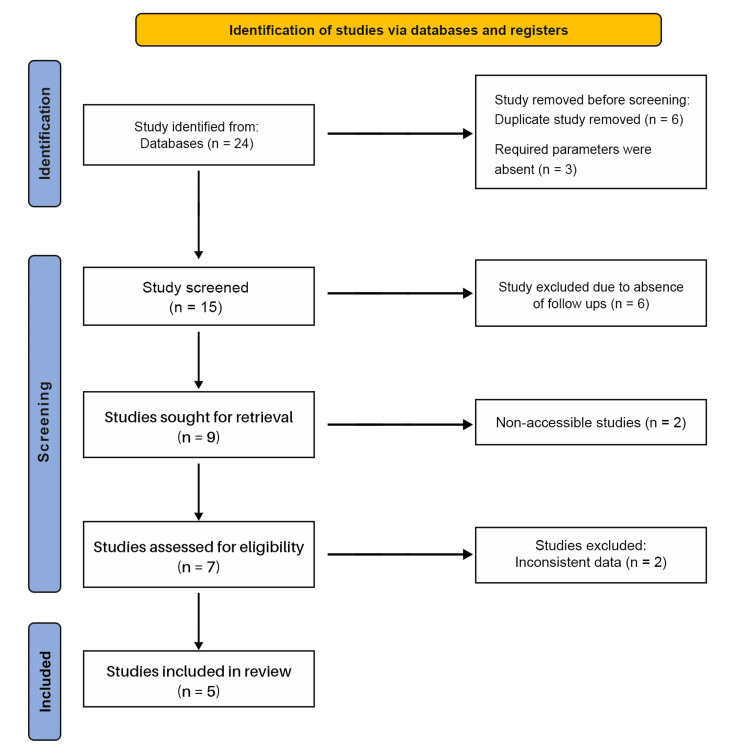
PRISMA Flowchart

The PRISMA flow for this review is as follows: 24 records were identified, six duplicates were removed, and three records lacking required parameters were excluded at the outset, leaving 15 records for screening. Of these, six were excluded due to the absence of clinical follow-up data. Nine studies were sought for retrieval but two were not accessible, leaving seven studies for eligibility assessment. A total of four studies were excluded: two were non-accessible and two had inconsistent data. Five studies were included in the final review. Figure 1 shows the PRISMA flow diagram of study selection for the review of prognosis of restored endodontically treated teeth by RTS.

Data Extraction

Two reviewers extracted the data independently using a standardized form that captured study characteristics (design, setting, sample size), participant/tooth characteristics (tooth type, age if available), details of the endodontic status and restorative intervention (direct composite without cuspal coverage versus crown/onlay, adhesive/cement protocol, post use and type), follow-up duration and completeness, definitions and measurements related to RTS, and outcomes with denominators at specified timepoints. Authors were contacted when clarification was required, if feasible. Any disagreements were resolved by consensus.

Risk-of-Bias Assessment

The randomized trials were appraised with the Risk of Bias 2 (RoB 2) tool, which produced judgments of low risk, some concerns, or high risk of bias across domains. The non-randomized comparative studies were appraised with ROBINS-I, with judgments of low, moderate, serious, or critical risk. Domain-level justifications were recorded, and overall study-level judgments were used to inform sensitivity analyses and the certainty of evidence.

Effect Measures and Synthesis

For the primary analysis, relative effects were expressed as risk ratios for fracture-free survival at approximately 24-36 months and 8-10 years using events and totals by arm when available. When only survival probabilities were reported, annual failure rates were derived using AFR = 1 − *S*^(1/t)^, where *S* is survival at *t* years. Hazard ratios were extracted when provided. Due to anticipated clinical and methodological heterogeneity, meta-analysis was considered only when at least two studies reported sufficiently comparable populations, interventions, RTS definitions, and timepoints; otherwise, findings were synthesized narratively with structured reporting of direction and magnitude of effect. Heterogeneity (when pooling was feasible) was assessed using random-effects models with inspection of clinical variability and statistical inconsistency.

Subgroup and Sensitivity Analyses

Planned subgroup comparisons examined how effects varied by RTS strata (one to three versus four to five surfaces lost; ferrule height and continuity; axial wall count), tooth type (anterior, premolar, molar), and post use/type (none, fiber, metal). Sensitivity analyses were planned to exclude studies at high/critical risk of bias and to assess the influence of follow-up duration and unit of analysis (tooth versus restoration level).

Certainty of Evidence

The overall certainty for key outcomes and timepoints was assessed using the Grading of Recommendations, Assessment, Development, and Evaluations (GRADE) approach, taking into account the risk of bias, inconsistency, indirectness, imprecision, and potential publication bias.

Ethics and Reporting

As this study synthesized previously published data, institutional review board approval was not required. Reporting followed PRISMA 2020 guidelines, and the study selection process is illustrated in the PRISMA flow diagram (Figure 1). Risk-of-bias assessments are summarized using RoB 2 and Risk Of Bias In Non-randomized Studies - of Interventions (ROBINS-I), with domain-level justifications provided.

Results

Study Selection

The database search identified 24 records. After removing duplicates (n = 6) and records without the required parameters (n = 3), 15 records remained for title/abstract screening. Six records were excluded due to the absence of clinical follow-up data. Nine full texts were sought, of which two were not accessible, leaving seven articles to be assessed for eligibility. Two were excluded for inconsistent or non-extractable data, resulting in five studies included in the review. The study selection process is depicted in Figure 1.

Study Characteristics

Five clinical studies met the inclusion criteria. These studies comprised one randomized controlled trial and four observational cohorts, with a total of 1,165 endodontically treated teeth. The randomized trial by Mannocci et al. [14] evaluated 117 premolars restored either with full-coverage crowns (n = 57) or non-cuspal-coverage resin composites (n = 60). A prospective cohort by Cagidiaco et al. [15] assessed 105 teeth (premolars/molars), of which 86 received crowns and 19 received resin composites. A large retrospective cohort by Dammaschke et al. [16] included 401 premolars/molars restored with crowns/bridges (n = 364) or resin composites (n = 37) and follow-up periods ranging from 60 to 192 months (mean 116.4 months). Suksaphar et al. [17] assessed 122 premolar teeth restored with resin composites or full-coverage crowns; survival was analyzed by restoration type and covariates including the number of occlusal contacts and surfaces lost. Landys Borén et al. [18] reported on 420 teeth comprising all tooth types; postoperative crowns were noted in 42.1% of cases, and 10-year survival was compared between crowned and non-crowned teeth. Where reported, posts were either not used, fiber posts, or cast posts; post usage varied by study and was generally higher in crowned teeth. Study characteristics are summarized in Table 1, including design, sample sizes by arm, follow-up, restoration type, post-use, and RTS descriptors.

**Table 1:** Characteristics of the included studies Numbers in parentheses indicate the number of teeth restored with the specified restoration type. ETT, endodontically treated teeth; RCT, randomized controlled trial

Study	Study Design	Year of Study	No. of Teeth	Type of Teeth	Restoration
Mannocci et al. [14]	RCT	2002	117	Premolar	Crown (57)
Cagidiaco et al. [15]	Prospective cohort	2007	105	Premolar/molar	Crown (86), resin composite (19)
Dammaschke et al. [16]	Retrospective cohort	2013	401	Premolar/molar	Crown/bridge (364), resin composite (37)
Suksaphar et al. [17]	Clinical trial	2018	122	Premolar ETT	Resin composite, full-coverage crowns
Landys Borén et al. [18]	Retrospective cohort	2014	420	Incisors (21.9%), canines (12.8%), premolars (28.7%), molars (36.6%)	Preoperative crown: 28.6%. Postoperative crown: 42.1%

Table 2 provides insight into the survival prognosis of different restoration types based on fracture criteria, survival rates, and follow-up periods.

**Table 2:** Survival prognosis of the included studies ETT, endodontically treated teeth

Study	Type of Post	Criterion of Survival from Fracture	Survival Rate Against Fracture		Follow-up Period (Months)
			Crown	Composite	
Mannocci et al. [14]	Prefabricated fiber post	Root fracture, post-fracture	100%	100%	12, 24, 36
Cagidiaco et al. [15]	Prefabricated fiber post	Post-fracture, vertical or horizontal root fracture	100%	100%	24
Dammaschke et al. [16]	Without a post, a prefabricated post, or a cast metal post	Fracture of the tooth and/or restoration	94%	91.90%	60–192 (mean: 116.4)
Suksaphar et al. [17]	Prefabricated fiber posts, cast metal posts, no post	Fracture analysis based on restoration type, number of contacts, sex, and surface losses	95.10%	77%	Not specified
Landys Borén et al. [18]	Post present in 12.1% of teeth	Tooth remained functional and not extracted due to root fracture	91.3% at 10 years (with crown restoration)	76% at 10 years (without crown restoration)	120 months (10 years)

As shown in Table 2, Mannocci et al. [14] reported that both crown and composite restorations had a 100% survival rate against fractures when using prefabricated fiber posts, with follow-up periods of 12, 24, and 36 months. Cagidiaco et al. [15] also used prefabricated fiber posts and reported similar 100% survival rates for both crowns and composites in cases of post-fracture, whether vertical or horizontal root fractures, with a 24-month follow-up. In contrast, Dammaschke et al. [16] found slightly lower survival rates of 94% for crowns and 91.9% for resin composite restorations, with a much longer follow-up period of 60-192 months (mean 116.4 months). Suksaphar et al. [17] analyzed fractures based on restoration type, number of contacts, sex, and surface losses; they found a survival rate of 95.1% for crowns but only 77% for resin composite restorations, although the follow-up period was not specified. Landys Borén et al. [18] observed a significant difference in survival rates between crown restorations (91.3% after 10 years) and non-crown restorations (76% after 10 years), with a follow-up period of 120 months.

Table 3 presents the restoration methods based on surface loss and specifically shows the percentage of teeth restored depending on the number of surfaces lost.

**Table 3:** Restoration of teeth based on their surface loss among the included studies *1–3 surfaces lost: refers to the loss of a combination of occlusal, proximal, or buccal/lingual surfaces; 4–5 surfaces lost: refers to significant loss of tooth structure involving multiple surfaces, often requiring full-coverage restoration.

Clinical Study	Total No. of Teeth (Resin Composite)	1–3 Surfaces Lost*	%	4–5 Surfaces Lost*	%
Mannocci et al. [14]	60	60	100.00%	0	0%
Cagidiaco et al. [15]	19	19	100.00%	0	0%
Dammaschke et al. [16]	37	31	83.80%	6	16.20%
Suksaphar et al. [17]	51	31	60%	20	39.21%
Landys Borén et al. [18]	Not reported				

As shown in Table 3, Mannocci et al. [14] and Cagidiaco et al. [15] reported no surface loss in the 19 and 60 teeth restored with resin composite, respectively, indicating a complete restoration. However, Dammaschke et al. [16] found that 37 teeth were restored with resin composites, with 83.8% of them having one to three surfaces lost, while the remaining 16.2% had four to five surfaces lost. The results obtained by Suksaphar et al. [17] indicated that 60% of the 51 teeth restored with resin composites had one to three surfaces lost, while the other 39.2% had four to five surfaces lost. Interestingly, Landys Borén et al. [18] did not report data related to surface loss in their study, which limits the conclusions regarding the specific restoration methods based on surface loss.

Risk of bias within studies

**Figure 2:**
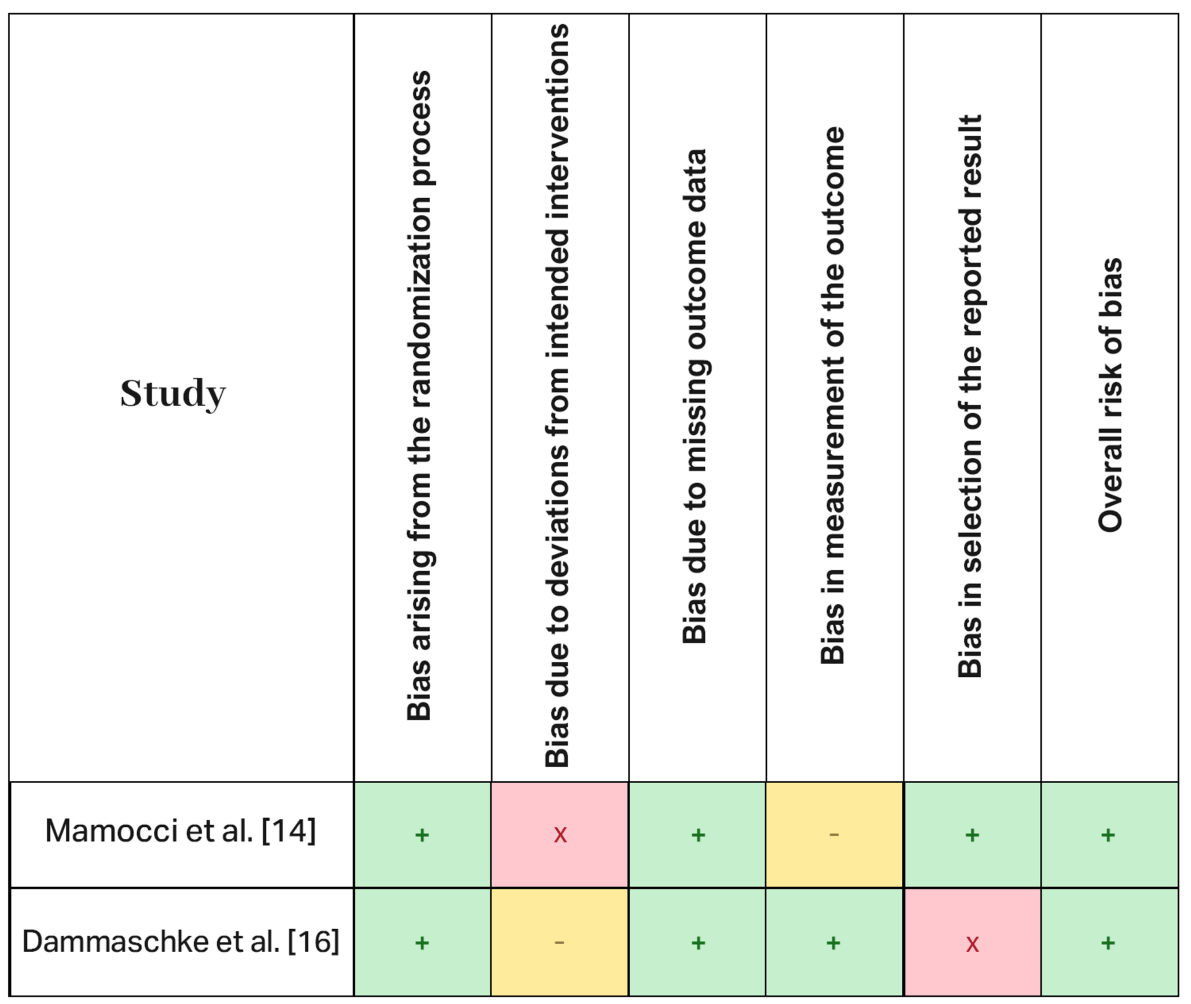
Bias assessment of the included studies using RoB 2.0 (randomized trials) Symbols: “+” (green) = low risk; “–” (yellow) = some concerns/unclear; “×” (red) = high risk.

**Figure 3:**
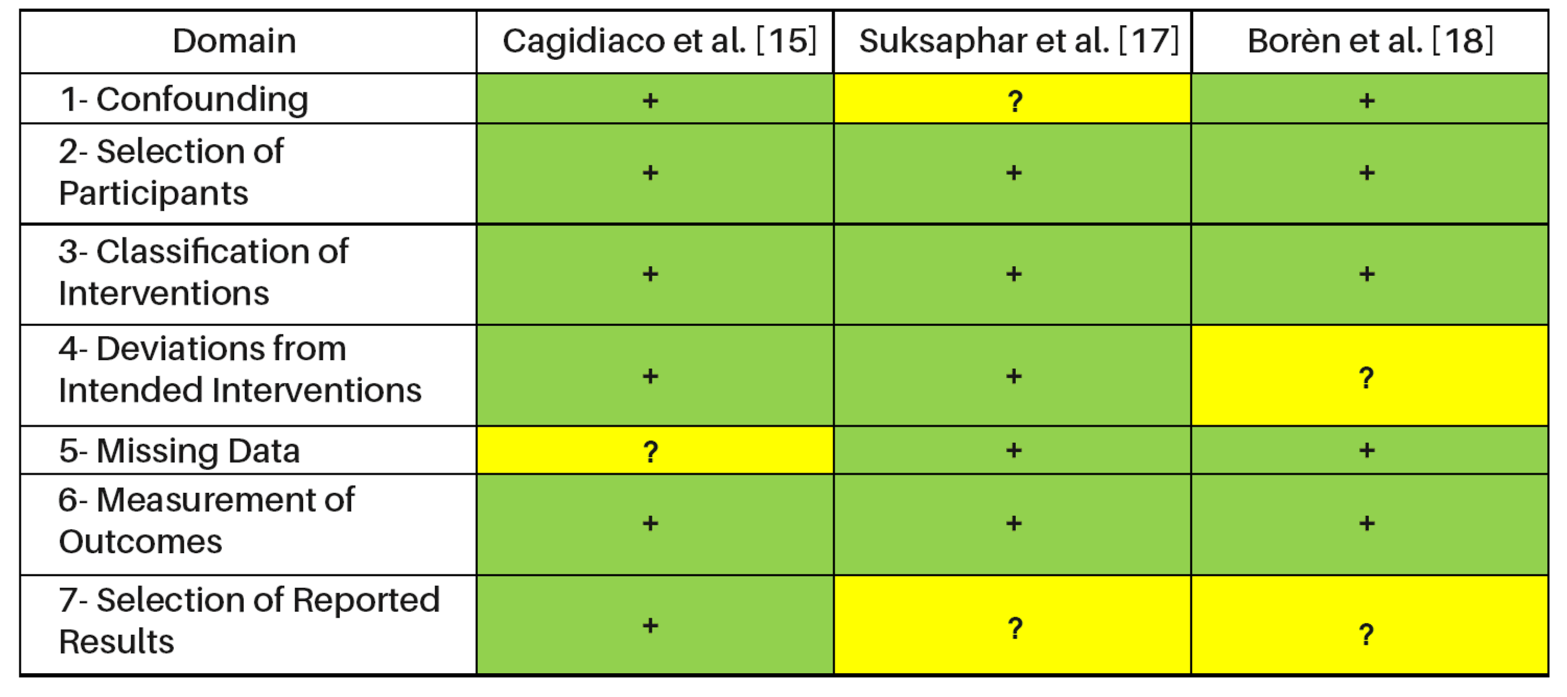
Bias assessment of the included studies using ROBINS-I (observational studies) Symbols: “+” (green) = low risk of bias; “?” (yellow) = some concerns/unclear risk of bias.

The single randomized trial [14] was generally considered low risk with some concerns in outcome measurement. The non-randomized studies [15-18] were judged at moderate overall risk of bias, primarily due to confounding, participant selection, and incomplete reporting of co-interventions (e.g., post use, adhesive/cement protocols). Risk-of-bias judgments are summarized in Figure 2 (RoB 2) and Figure 3 (ROBINS-I), with domain-level justifications provided.

As shown in Figure 2, the bias assessment of the included studies reveals varying levels of risk across different domains. Mannocci et al. [14] exhibited low bias in randomization and missing outcome data, but they had high bias due to deviations from intended interventions and unclear bias in the measurement of outcomes. Despite these concerns, the overall risk of bias was considered low. Dammaschke et al. [16] also had low bias in randomization, missing data, and outcome measurement, but they showed unclear bias due to deviations from intended interventions and high bias in the selection of reported results, resulting in a moderate overall risk of bias.

As shown in Figure 3, the ROBINS-I risk of bias assessment for Cagidiaco et al. [15], Suksaphar et al. [17], and Borén et al. [18] reveals some differences across domains. Cagidiaco et al. and Borén et al. were classified with low risk (+) for confounding, participant selection, and intervention classification, while Suksaphar et al. was judged at moderate risk (?). For deviations from intended interventions, Cagidiaco et al. and Suksaphar et al. were rated low risk, but Borén et al. showed moderate risk (?). Missing data was flagged as moderate risk for Cagidiaco et al., whereas the other two studies had low risk. All three studies demonstrated low risk in measurement of outcomes. However, both Suksaphar et al. and Borén et al. were judged at moderate risk (?) for selective reporting, indicating potential concerns regarding outcome reporting in these studies.

Primary outcomes: fracture-free tooth survival

Short-term outcomes (~12-36 months) were uniformly high across interventions. Mannocci et al. [14] reported 100% fracture-free survival at 12-36 months in both the crown and composite groups. Cagidiaco et al. [15] similarly reported 100% survival at 24 months for both restorations. Longer-term outcomes showed divergence favoring cuspal coverage. Dammaschke et al. [16] observed 94% survival for crowns and 91.9% for resin composites at a mean of 116.4 months. Suksaphar et al. [17] reported 95.1% survival for crowns and 77% for resin composites (follow-up period not uniformly specified). Landys Borén et al. [18] found that at 10 years (120 months) survival was 91.3% with crowns versus 76% without crowns. Taken together, these data suggest equivalence in the short term but a clinically relevant advantage for crowns at longer follow-up horizons.

Secondary outcomes and failure patterns

Where specified, the survival criterion was tooth-level fracture avoidance (root or catastrophic fracture) rather than restoration retention alone [14-16]. Landys Borén et al. [18] defined survival as the tooth remaining functional and not extracted due to root fracture; failure rates over 10 years were higher in teeth without cuspal coverage. Detailed failure modes (restorable vs. catastrophic) were variably reported; fiber-post use was associated with fewer catastrophic failures under incisal loading in studies that discussed post material properties. Although comparative, adjusted analyses were limited.

Remaining Tooth Structure and Effect Modification

Reporting of RTS was inconsistent across studies, limiting formal subgroup synthesis. Nevertheless, where data were available for resin composite groups, cases predominantly involved less extensive loss of coronal structure. Studies by Mannocci et al. [14] and Cagidiaco et al. [15] involved resin-composite cases where the teeth had one to three surfaces lost. In Dammaschke et al. [16], 83.8% of resin-composite cases involved one to three surfaces lost and 16.2% involved four to five surfaces lost. In Suksaphar et al. [17], 60% of resin-composite cases had one to three surfaces lost and 39.21% had four to five surfaces lost; Landys Borén et al. [18] did not report surface-loss strata. No study provided fracture outcomes stratified by both restoration type and identical, pre-specified RTS categories with sufficient detail to allow comparative pooling. The available distributions indicate a tendency for resin composites to be used in teeth with less severe structural loss. This allocation pattern could bias unadjusted comparisons in favor of composites in the short term. Despite this, long-term survival favored crowns where RTS was more extensively compromised.

Synthesis of Results

Planned meta-analysis was not performed due to heterogeneity in study designs, follow-up durations, outcome definitions, and incomplete reporting of RTS strata and denominators at common timepoints. Instead, a structured narrative synthesis was conducted. Across ~24-36 months, both crowns and non-cuspal-coverage composites demonstrated high fracture-free survival (~100% in the two studies reporting these windows) [14, 15]. At ~8-10 years, studies consistently reported higher survival for crowned teeth, with absolute differences of ~13-19 percentage points relative to non-crowned/composite restorations [16-18]. These patterns align with the biomechanical expectation that cuspal coverage becomes increasingly protective as structural loss accumulates and time under load increases.

Certainty of Evidence

Based on GRADE scoring, the certainty for short-term equivalence between crowns and resin composites was judged low to moderate due to imprecision and limited events. The certainty supporting a long-term survival advantage for crowned teeth was rated low to moderate, downgraded for risk of bias in non-randomized evidence and inconsistency in RTS reporting. Table 4 summarizes the main outcomes of this review with their certainty of evidence according to the GRADE approach.

**Table 4:** GRADE summary of findings: prognosis of endodontically treated teeth restored with direct composites versus crowns Catastrophic/root fracture refers to a non-restorable tooth/root fracture typically resulting in extraction. GRADE certainty symbols: ⬤⬤⬤⬤ = High, ⬤⬤⬤◯ = Moderate, ⬤⬤◯◯ = Low, ⬤◯◯◯ = Very low. AFR, annual failure rate; CI, confidence interval; Cohorts, prospective or retrospective observational studies; GRADE, Grading of Recommendations, Assessment, Development, and Evaluations; RCT, randomized controlled trial; RST, remaining tooth structure

Outcome	Time Frame	Effect (Direction and Magnitude)	No. of Studies (Teeth)	Study Design	Certainty (GRADE)	Reasons for Rating
Fracture-free tooth survival (short-term)	≤36 months	No important difference between crowns and direct composites (both ~100% survival in two studies)	2 studies (~222 teeth)	RCT + cohort	⬤⬤◯◯	Downgraded for imprecision (few events) and inconsistency (small, heterogeneous cohorts)
Fracture-free tooth survival (long-term)	8–10 years	Favors crowns (absolute difference ~13–19 percentage points vs. no crown/composite)	3 studies (~943 teeth)	Cohorts	⬤⬤◯◯	Downgraded for risk of bias (non-randomized), inconsistency (RTS reporting varies), possible confounding
Catastrophic/root fracture	5–10 years	Trend towards fewer catastrophic fractures with crowns in structurally compromised teeth	2 studies (~400 teeth)	Cohorts	⬤⬤◯◯	Downgraded for imprecision (sparse events) and indirectness (definitions vary)
Restoration survival/need for re-treatment	2–10 years	No consistent difference (high short-term survival; attenuation by 10 years in both groups)	3 studies (~500 teeth)	RCT + cohorts	⬤⬤◯◯	Downgraded for inconsistency (follow-up, protocols) and imprecision
Effect modification by RTS	2–10 years	1–3 surfaces lost / preserved walls: composites perform well. 4–5 surfaces lost / disrupted ferrule: crowns superior	3–4 studies (subgroup info)	Cohorts	⬤◯◯◯	Downgraded for indirectness (non-uniform RTS categories), inconsistency and imprecision (no pooled stratum-specific effects)

Discussion

Summary of Evidence

The present analysis demonstrates that the remaining coronal tooth structure is the most influential factor determining the long-term prognosis of endodontically treated teeth. Multiple in vitro and clinical investigations corroborate a strong correlation between the volume and configuration of residual tooth structure and both fracture resistance and survival outcomes [1,2]. Notably, endodontic treatment alone does not render teeth brittle; material and mechanical testing has shown that endodontically treated teeth retain comparable toughness and fracture resistance to vital teeth. While there are minor differences in hardness, these are unlikely to influence clinical outcomes [1].

Firstly, biomechanical studies reveal that structural integrity, particularly in the cervical region, dramatically influences stress distribution under masticatory loads. The ferrule effect, involving a circumferential band of sound dentin ≥1.5-2.0 mm in height, is repeatedly validated as critical to resist vertical and lateral loading [2,8,9]. Juloski et al. [8] established that an incomplete ferrule is better than none and that a full ferrule yields the highest fracture resistance [8]. Sorensen and Engelman [9] confirmed that teeth with intact axial walls and a ferrule had significantly greater fracture resistance than those without [9]. Therefore, preserving the coronal rim and axial dentin should be a non-negotiable goal during both endodontic access and restorative preparation. Clinically, ferrule continuity and wall location are as pertinent as height; even with similar nominal ferrule heights, outcomes can be influenced by interproximal discontinuities and thin dentin (≤1 mm) [8,9].

Secondly, clinical evidence supports laboratory findings. In a large-scale multicentre study, mortality of endodontically treated teeth was lowest when full-coverage restorations and intact proximal contacts were present [3,17]; this highlights the impact of coronal coverage and contact preservation. Practice-Based Research Network data reveal a ∼13.9% restorative failure rate at three to five years, with intracoronal restorations failing more often than crowns [3,17]. Survival of endodontically treated teeth is significantly improved if definitive crowns are placed early post-endodontic treatment, as delayed coverage increases the extraction danger [16-18]. There is also a potential allocation effect: intracoronal restorations are administered on teeth with lesser structural loss, whereas cuspal coverage is given to more compromised teeth. Due to the lack of adjusted analyses, unmeasured confounding may have an effect on basic comparisons [3].

Endodontically treated teeth lacking cuspal coverage are more likely to fail over longer follow-up, especially when four to five or more surfaces are lost [14-18]. Survival rates for endodontically treated teeth with full crowns or bonded restorations reach ~94-100%, compared to lower survival with intracoronal restorations [14-18]. Furthermore, several cohorts have reported absolute survival gaps across ~8-10 years. Consistent with time-under-load fatigue effects, this occurs on the order of a 13-19% absolute survival advantage for cuspal coverage [14-18]. Importantly, survival is tied to structural conservation: greater remaining tooth volume and robust contact points are directly associated with improved prognosis [11,17].

Interpretation and Clinical Implications

The role of posts in survival and fracture patterns is nuanced. While root-canal-treated teeth often require posts to retain core material, posts should never substitute for a ferrule. Fiber posts, with mechanical properties such as dentin, distribute stress favorably and reduce catastrophic root fractures compared to metal posts [2,12]. However, clinical evidence remains ambiguous: some meta-analyses suggest that post placement improves survival but does not necessarily compensate for the absence of structural reinforcement [11]. Excessive post-space preparation can also weaken roots and jeopardize coronal seal, underlining the need for prudent selection and minimal invasiveness [3,11]. In clinical practice, fiber posts should be reserved for retention in severely reduced cases, such as those with 0-1 axial wall. Routine post placement should be avoided, especially if core retention is already provided by a continuous ferrule and adequate coronal dentin [9-12].

The timing and quality of coronal restoration are also critical. A poor coronal seal is closely linked to periapical failures; conversely, definitive crowns following good endodontic therapy are associated with better outcomes [2,16-18]. Immediate or prompt restoration within a few months significantly reduces failure risk, reinforcing the need for integrating endodontic and restorative planning [2,16-18]. In terms of tooth level, a durable coronal seal is for beneficial for long-term function than specific post systems when there are adequate structures and ferrule [2,11].

Beyond ferrule and posts, other factors such as proximal contacts, tooth type, and periodontal health emerge as significant. Teeth with intact proximal contacts show enhanced stability and longevity, likely due to shared occlusal loads and reduced lateral stress [17]. Molars tend to outperform premolars and anterior teeth in survival metrics, which could be due to root morphology and less frequent need for intracoronal restorations [3]. Periodontal disease may compound mechanical weakness, stressing the need for a holistic periodontal-restorative endodontic approach [10,13]. Future comparative studies should prospectively evaluate occlusal scheme, contact integrity, and periodontal status, as these confounders are inconsistently reported yet significantly affect outcomes [3,17].

The literature also discusses the merits of minimally invasive endodontics and conservative restorative methods. Preserving coronal and radicular dentin during access and shaping enhances structural resilience. To promote tissue preservation and restorative longevity, clinicians should adopt adhesive protocols, avoid unnecessary bevels, and use conservative crowns or onlays [11,12]. In cases of suboptimal ferrule, crown lengthening or orthodontic extrusion may restore structural prerequisites for a durable restoration [2,13]. If feasible aesthetically, establishing a circumferential ferrule ≥1.5-2 mm can transform the restorative trajectory from guarded to favorable and foster more favorable long-term function [2,8,9].

Preserving the coronal tooth structure should remain a top priority in the clinical management of endodontically treated teeth [14]. It is crucial to prioritize early restoration, with full-coverage crowns recommended for teeth with four or more surface loss to ensure adequate fracture resistance and long-term success [15]. For cases with less than four surface losses (one to three surfaces), minimally invasive techniques, such as direct resin composite restorations, can be used effectively to restore the tooth while preserving healthy tooth structure. Minimally invasive techniques in the context of endodontically treated teeth focus on restoring the tooth structure while preserving as much healthy tissue as possible. This approach prioritizes preserving the coronal tooth structure to avoid further weakening the tooth, which is critical for maintaining its function and longevity [14,15]. Direct resin composite restorations are a prime example of minimally invasive methods used for teeth with one to three surfaces lost. As they bond directly to the tooth, these restorations provide a resilient and effective solution by restoring tooth integrity without having to extensively remove the RTS [16]. The ferrule effect is also crucial in the restoration of endodontically treated teeth. It refers to a circumferential band of healthy tooth structure (typically 2-4 mm of dentin) that remains around the core of the tooth after preparation [16,17]. This band of sound dentin enhances the strength of the tooth and its ability to withstand functional forces. The ferrule effect is particularly important in preventing root fractures and improving the retention of the restorative material, thereby enhancing the overall prognosis of the tooth [17]. Furthermore, clinicians should aim at maintaining the ferrule effect by preserving a circumferential band of sound dentin, as this significantly enhances the tooth’s ability to withstand functional loads and resist fractures. A concise comparison of surface loss in endodontically treated teeth reveals that teeth with one to three surfaces lost generally respond well to direct resin composite restorations [16,17]. These restorations provide adequate fracture resistance and long-term survival when the remaining coronal structure is preserved. In contrast, for teeth with four to five surfaces lost, full-coverage crowns are needed to ensure sufficient fracture resistance and survival. This is because teeth with extensive structural loss are more vulnerable to fracture and therefore require more comprehensive coverage to prevent failure [17,18]. If there are conflicts in adopting these recommended thresholds for one to three vs. four to five surfaces, ferrule continuity should be prioritized over surface count. For example, a tooth with fewer surfaces but an interrupted ferrule may still benefit from cuspal coverage [16,17]. A comparative analysis of surface loss in endodontically treated teeth highlights distinct differences in the restoration approach based on the extent of tooth structure lost. For teeth with one to three surfaces lost, direct resin composite restorations are often sufficient to restore function and maintain long-term survival [15,17]. These restorations are minimally invasive, preserving most of the remaining healthy tooth structure while providing adequate fracture resistance. Studies have shown that resin composites can effectively bond to the tooth, offering durability and resistance to fracture when there is a sufficient amount of remaining coronal structure [12]. The ability to preserve healthy tissue while restoring tooth function makes resin composite restorations a suitable choice for cases of minimal surface loss. In contrast, teeth with four to five surfaces lost are significantly more vulnerable to fracture due to the substantial loss of tooth structure. In these cases, full-coverage crowns are essential for providing adequate fracture resistance and ensuring the tooth’s long-term survival. The loss of a large portion of the tooth makes it more susceptible to stress and functional loading, which can lead to failure if not properly restored [14,17,18]. With full-coverage crowns, the functional forces are distributed more evenly across the tooth; this improves overall strength and resistance to fracture, particularly when structural loss is extensive [14,15,17,18]. By providing a protective barrier, these crowns reduce the risk of further damage. Therefore, while minimal surface loss can be managed using direct resin composite restorations, more extensive loss generally requires cuspal coverage [14,15,17,18]. That said, most current evidence is observational and has only low-to-moderate certainty. The choice of treatment should therefore be individualized to the tooth’s RTS profile and occlusal environment [14,15,17,18].

One possible issue with this systematic review is potential publication bias, as studies with positive findings (such as high survival rates for crowns or resin composites) are more likely to be published, while studies with negative or inconclusive results might remain unpublished. This bias could lead to an overestimation of the effectiveness of the restorative treatments. A thorough search strategy that included both grey literature and manual searches was employed to minimize this risk. However, the absence of long-term prospective randomized controlled trials may contribute to uncertainty in the overall conclusions. Furthermore, the effects of small study sizes and selective reporting cannot be excluded, and inconsistent RTS definitions likely obscure true effect modification. For future research, standardized RTS reporting for factors such as axial wall count, surfaces lost, and ferrule height/continuity would significantly improve the comparability of findings.

Limitations

This systematic review has several limitations that must be considered when interpreting the results. First, the heterogeneity in study designs, including randomized controlled trials, cohort studies, and clinical trials, may introduce variability in the findings. Additionally, the small sample sizes in some of the studies limit the generalizability of the results. Another important limitation is the lack of long-term prospective randomized trials to provide more robust data on the long-term survival of different restoration types. Finally, variability in the restoration types used (such as different resin composites and crowns) and the extent of tooth surface loss across studies adds complexity to the direct comparison of results. Other limitations include varying outcome definitions (tooth-level vs restoration-level) and unit-of-analysis issues (multiple teeth per patient), which can bias precision and hinder pooling. For these reasons, a quantitative meta-analysis was not feasible, as differences in study design, outcome definitions, follow-up duration, and reporting of RTS prevented meaningful statistical synthesis. To overcome these issues, future trials should specify common timepoints and adjust for key confounders including tooth type, contact integrity, post use, and time to definitive restoration [3,16-18]. In clinical practice, the following actions need to be prioritized: preserve the dentin, establish or augment a circumferential ferrule ≥1.5-2 mm where feasible, provide timely cuspal coverage when structural loss is substantial, and reserve posts for retention rather than reinforcement [2,8,9,11,12].

Future Research Directions

To overcome these issues, future trials should specify common timepoints and adjust for key confounders including tooth type, contact integrity, post use, and time to definitive restoration [3,16-18]. Standardized reporting of RTS (axial wall count, surfaces lost, ferrule height/continuity) is needed. Large, prospective randomized controlled trials are required to clarify whether minimally invasive direct restorations can truly match cuspal coverage in structurally compromised teeth.

## Conclusions

The study concluded that both crowns and resin composites provide high survival rates, but crowns generally demonstrate better long-term outcomes. While surface loss varied across studies, crowns generally performed better in minimizing this. Overall, most studies exhibited a low risk of bias, though some showed deviations from intended interventions and outcome measurements. This systematic review emphasizes that the preservation of coronal tooth structure is the most decisive factor affecting the prognosis of endodontically treated remaining teeth. While both full-coverage crowns and non-cuspal coverage resin composite restorations established high survival rates, teeth with limited tooth structure loss (one to three surfaces) can be effectively restored with direct composite without significantly compromising survival. However, for cases with extensive structural loss (four to five surfaces), full-coverage crowns are suggested to confirm resilient fracture resistance and lasting success. Careful case selection, adherence to the principles of minimally invasive dentistry, and the maintenance of a ferrule effect are critical for optimal outcomes.
